# Fractionation of waste-derived volatile fatty acids by multi-stage adsorption using activated charcoal and Diaion HP-20 resin

**DOI:** 10.1080/21655979.2025.2458366

**Published:** 2025-02-04

**Authors:** Negar Basereh, Steven Wainaina, Amir Mahboubi, Mohammad J. Taherzadeh

**Affiliations:** Swedish Centre for Resource Recovery, University of Borås, Borås, Sweden

**Keywords:** Waste-derived volatile fatty acids, Adsorption, Fractionation, activated charcoal, Diaion resin

## Abstract

Substituting waste-derived Volatile Fatty Acids (VFAs) with their conventionally applied fossil-derived counterparts in a spectrum of industrial applications necessitates its proper fractionation into individual acids. This study explored a multi-stage batch adsorption approach for fractionating acidogenic fermentation VFAs effluents from food waste (FW) and chicken manure (CKM) using Diaion HP-20 and activated charcoal. Initial screening at different washing conditions and pH (3.5 and 6.5) revealed the unwashed granular-activated charcoal (GAC-Unwashed) and milli-Q water-washed Diaion (DI-MQ Washed) as the most promising candidates for VFA fractionation of a synthetic VFA mixture at 4 gL^−1^. At pH 3.5 ($<pK_{a}$), GAC-Unwashed adsorbed 2–6 carbon atom VFAs completely, while DI-MQ Washed exhibited minimal adsorption of acetic acid (AA) (8%), favoring caproic (CA) and valeric acids (VA) (>97%). While at pH 6.5 $(>pK_{a}$), GAC-Unwashed selectively targeted VA (79%) and CA (100%). Fractionating VFAs from FW and CKM were conducted in a two-stage adsorption process with optimal results being achieved using GAC-Unwashed at FW initial pH (5.3) and DI-MQ Washed at pH below CKM $pK_{a}$ (3.5), respectively. The first adsorption stage primarily adsorbed higher molecular weight (MW) VFAs (FW:99.1% CA, CKM:72.9% butyric acid (BA)) with a minor quantity of lower ones (FW:56.5% BA, CKM:29.3% propionic acid (PA)), leaving AA intact. Subsequent stages aimed to isolate AA by adsorbing the remaining low MW VFA (FW:58.9% BA, CKM:27.8% PA, 70% BA) other than AA, indicating effluent fractionation while preserving and purifying AA. Applied selective multi-stage adsorption approach offers a promising method to broaden waste-derived VFA applications.

## GRAPHICAL ABSTRACT

**Figure:**
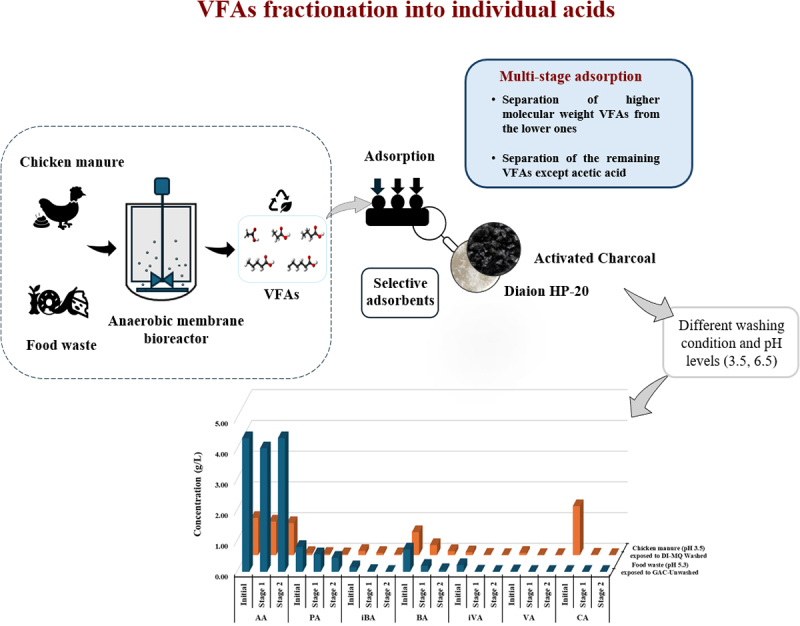

## 1. Introduction

Volatile fatty acids (VFAs), a group of valuable short-chain carboxylic acids ranging from two to six carbon atoms, are important building block chemicals with a substantial growing market appeal [1,2]. The largest global VFA market share is dominated by acetic, propionic, and valeric acid, projected to reach 23.02, 2.2 [3,4] and 24.3 billion USD by 2030 [5], respectively. The notable market growth of VFAs finds its roots in several industrial applications across diverse sectors, including chemical, petrochemical, agricultural, pharmaceutical, textile, food-beverage, and water and wastewater treatment [1,6–9].

VFAs are conventionally derived from fossil resources, leading to high greenhouse gas emissions [1,7,8,10]. However, producing VFAs from renewable resources is a potential game-changer, capable of converting linear to the circular economy by having a small carbon footprint and high environmental benefits [1,6,7,9]. Sustainable production of VFAs can be achieved through biological methods including pure culture fermentation and anaerobic digestion (AD) [1]. AD is a widely adopted technology for the treatment and valorization of organic wastes including, but not limited to, food waste, agriculture residues, manure, and municipal solid waste [6,9,11–13]. While biogas is the primary AD product, VFAs – the intermediate products generated during the acidogenesis and acetogenesis steps – have recently gained much more interest due to their high unit value and versatile applications [1,2,12]. By halting the methanogenesis step, the AD process can be adapted for VFA production and accumulation, offering higher kinetic rate and potentially lower capital costs compared to traditional AD [13,14]. This altered process is referred to as acidogenic fermentation or arrested anaerobic digestion (AAD) [13].

The product of AAD varies based on the fermentation approach and waste source, resulting in a dilute mixture of short-chain aliphatic mono-carboxylates (VFAs), ions, trace elements, organic acids, alcohols, residual carbohydrates, microorganisms, and proteins [2,15,16]. The recovered AAD mixed VFA effluent can be utilized in different applications like the production of bioenergy, biofuels, bioplastics (PHA), medium-chain fatty acids, while also acting as a carbon source in biological nutrient removal (BNR) or alternative feed ingredients for sustainable ruminant feed [1,6,8,9,17–19]. However, the mixed VFA effluent is deemed less valuable compared to the pure form of individual acids [8]. Therefore, substituting fossil-derived VFAs with waste-derived VFAs for comparable industrial applications necessitates efficient downstream processing of AAD mixed VFA effluent including concentration, purification, and fractionation into individual acids. Fractionation appears to be the most challenging due to the similarities in the physicochemical properties of VFAs, complexity, and the low concentration of AAD effluents. For this reason, most VFA recovery methods have primarily focused on the total VFA separation rather than fractionation into individual acids [6,20].

Affinity separation is a potential technique for the selective recovery of the desired VFA from a dilute complex matrix, wherein the affinity agent targets specific VFA molecules [15,21]. VFA affinity techniques encompass adsorption [10,15,20–25], liquid–liquid extraction [26–28], membrane contactors filled with extractants [29], liquid-membranes [30–32], and solvent impregnated resins [33]. Among these methods, adsorption is a well-established, economically viable, easily operable, and efficient VFA separation method [6,12,23,24]. Key concerns with adsorption include adsorbent regeneration, desorption efficiency, and co-adsorption of available ions and molecules in the effluent [34]. Selective adsorption of VFA species can be accomplished by selecting an appropriate adsorbent defined by adsorbent surface properties and operating conditions like adsorbent dosage, pH, and temperature. The adsorption pattern is significantly influenced by the nature of the acids and solution’s pH [9,35].

Researchers have investigated various adsorbents for VFA recovery, primarily focusing on maximizing the separation of all VFAs present in the solution [10,15,20,23,35–38]. Studied adsorbents consist of resin-based [10,15,20,23,35–37], activated carbon [24,36,37], zeolite [22,39], superparamagnetic porous adsorbents [21], and hybrid magnetic molecularly imprinted polymers [25,40]. A review of the pertinent studies reveals that while weak base anion tertiary amine generally exhibit the highest recovery of all VFAs [10,20,23,36,37], non-functionalized amine resin demonstrated superior performance when simultaneous purification and separation are required [15]. Besides, greater affinity to VFAs with higher molecular weight (MW) or longer chain length has been observed where the hydrophobic interaction prevails [9,15,21,23,35]. Specifically, non-functionalized resins and activated carbon have shown particular promise in selectively adsorbing higher MW VFAs [9,15,20,35,36]. Notably, Diaion HP-20 resin could barely adsorb acetic acid from a mixture of VFAs containing 2–5 carbon atoms while it adsorbed approximately 5% of propionic acid, 29% of each of isobutyric and butyric acids, 66% of valeric acid, and 74% of caproic acid [20]. This highlights the suitability of Diaion HP-20 resin for VFA fractionation due to its high selectivity and lack of co-adsorption, while activated carbon emerges as another promising, cost-effective alternative for this purpose.

Given the distinct selectivity of adsorbents like activated carbon and Diaion HP-20 resin, for different VFA components, fractionation of VFA mixture can be achieved through selective multi-stage adsorption. This concept involves each stage having a specific fractionation goal, achieved through the selection of suitable adsorbents under optimized operational conditions. In this novel approach, initial stages aim to separate higher molecular weight VFAs from lower ones, while subsequent stages focus on removing remaining VFAs and purifying acetic acid. While selective desorption of binary and tertiary VFAs mixtures has been studied in limited studies [15,24], only one study briefly addressed the potential of sequential adsorption employing two resins (Amberlite IRA-67 and Dowex Optipore L-493) for the selective separation of acetic acid from a synthetic VFA mixture of 2–5 carbon atoms [20]. To the knowledge of the authors, no previous study has focused on VFA mixture fractionation of AAD VFA effluent using selective multi-stage adsorption. Systematically exploring this approach to separate and recover individual VFAs from waste-derived effluents enhances the efficiency of VFA recovery processes and enables their substitution for fossil-derived counterparts in biorefinery operations.

This study aimed to fractionate VFAs produced through acidogenic fermentation using multi-stage adsorption with Diaion HP-20 resin and activated charcoal. Different factors, including adsorbent washing and pH, were initially studied in a synthetic VFA mixture to identify suitable adsorbents and optimal fractionation conditions. Subsequently, the viability of the selected adsorbents was investigated for fractionating two different acidogenic fermentation effluents derived from chicken manure and food waste using various scenarios through a two-stage adsorption process, with results compared to the corresponding synthetic mixtures.

## 2. Materials and methods

### 2.1. Materials

The adsorbents used in the present study were Diaion HP-20 resin (DI) (particle size: 250–850μm), Powdered Activated Charcoal (PAC) (particle size < 100 μm (∼90%)), and Granular Activated Charcoal (GAC) (extra pure, particle size 1.5 mm), all obtained from Merck Supelco (Darmstadt, Germany). VFAs including acetic acid (AA, ≥99.7%), propionic acid (PA, ≥99.5%), isobutyric acid (iBA, 99%), butyric acid (BA, ≥99%), isovaleric acid (iVA, 99%), valeric acid (VA, ≥99%), caproic acid (CA, ≥99%), as well as methanol (gradient grade, ≥99.9%) were purchased from Sigma-Aldrich (MO, USA). For pH adjustment, HCl (Sigma-Aldrich, ACS reagent, 37%) at a concentration of 6 M and NaOH (Sigma-Aldrich, reagent grade, ≥98%) at a concentration of 12 M were prepared.

### 2.2. Fractionation of volatile fatty acids from the food waste (FW) and chicken manure (CKM) derived VFA effluent

#### 2.2.1. Pre-adsorption washing

The adsorbents were washed to remove the contaminants and impurities while enhancing the active sites on the solid matrix without altering the chemical surface structure. Therefore, GAC and PAC were solely washed with milli-Q water (MQ), while DI underwent two distinct washing processes: 1) methanol and milli-Q water according to the supplier’s procedure, 2) milli-Q water.

The washing procedure with milli-Q water involved placing 3% (w/v) adsorbents in a flask containing milli-Q water on a rotary shaker incubator (200 rpm and 25 °C) for 2-hour intervals where the washed liquid reached a constant pH. The washed samples were denoted by PAC-MQ Washed, GAC-MQ Washed, and DI-MQ Washed. While the washing procedure of DI with methanol and milli-Q water was as follows: DI was transferred to a beaker and a sufficient amount of methanol (gradient grade, ≥99.9%) was added to cover the surface by 2.5–5 cm. The mixture was stirred gently for 2 min and allowed to rest for 15 min. Next, the methanol was decanted, replaced with milli-Q water, stirred, and remained for 10 min. The washed sample was denoted by DI-MQ+ME Washed. Following the washing process, the washed activated charcoal was dried overnight in the oven at 70°C, while DI was air dried overnight at room temperature.

#### 2.2.2. Adsorbent Screening

**Figure 1.**
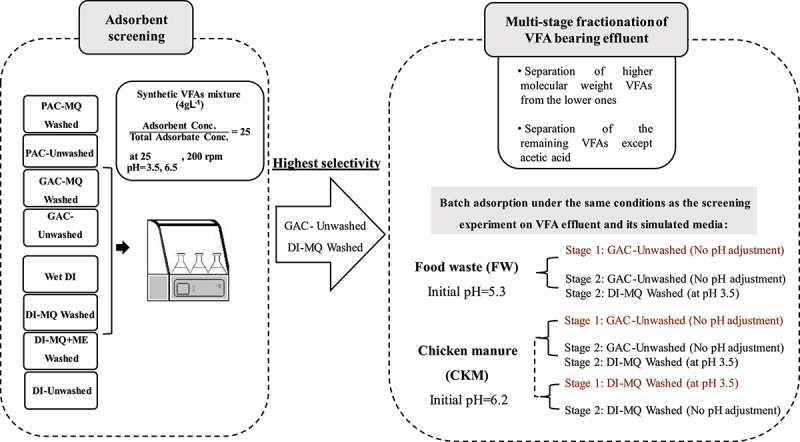
Schematic of the experimental procedure used in this study (PAC: Powdered Activated Charcoal, GAC: Granular Activated Charcoal, DI: Diaion HP-20, MQ: Milli-Q water, ME: Methanol).

Batch adsorption experiments were conducted to screen the effective adsorbents, assessing the impact of washing and pH on adsorption performance in a synthetic VFAs mixture. Both washed and unwashed adsorbents were tested under two distinct pH conditions: a) pH 3.5, below the $pK_{a}$ of all VFAs, and b) pH 6.5, above the $pK_{a}$ of all VFAs. This approach aimed to explore how the chemical state of VFAs influences adsorption efficiency. The schematic of the experimental procedure is presented in Figure 1.

The experiments were conducted in a rotary shake incubator (New Brunswick^TM^ Excella® E 24, Hamburg, Germany) at 200 rpm and 25 ± 2°C. Each of the 100 mL Erlenmeyer flasks contained 30 mL VFAs media synthetic VFA mixture with 3 g of the adsorbent (washed/unwashed) at two initial pH of 3.5 and 6.5. The synthetic VFAs mixture had a total initial concentration of 4 gL^−1^ comprising five main VFAs, each with a concentration of 0.8 g L^−1^. The adsorbent dosage-to-adsorbate initial concentration ratio was maintained at 25 (g L^−1^ per g L^−1^). The conditions were selected according to the procedure described in Eregowda et al. [20]. The liquid-phase samples were collected at intervals of 10, 20, 30, 60, 120, and 240 min and filtered through 0.2 µm syringe filter to monitor their respective pH and VFA concentrations. Each adsorption experiment continued till reaching the equilibrium. All screening experiments were performed in triplicate.

#### 2.2.3. Multi-stage VFAs fractionation from synthetic and acidogenic fermentation effluents

Based on the results of the screening experiment, effective adsorbents and optimal experimental condition for VFA fractionation were identified. Subsequently, fractionation of two types of effluents and corresponding synthetic VFAs mixtures was performed through a two-stage batch adsorption experiment.

VFA effluents were derived from acidogenic fermentation of chicken manure (CKM) and food waste (FW) digested in membrane bioreactors as described in [41] and [42], respectively. The concentrations of VFA and the initial pH in these effluents are given in Table 1. Synthetic VFA mixtures were prepared by mimicking the concentrations of VFAs in each respective effluent, and their concentrations closely matched those presented in Table 1. The interest in these effluents lies in their distinct VFAs distribution. CKM was rich in lower MW VFAs, particularly AA, with PA and BA present at intermediate levels. Conversely, in FW, both higher and lower MW VFAs were dominant, notably CA and AA.

**Table 1.** VFAs concentration and pH of initial chicken manure (CKM) and food waste (FW) derived VFA bearing effluents in the first adsorption stage.

VFA source	VFA Concentration ± SD (g L^−1^)							pH
	AA*	PA*	iBA*	BA*	iVA*	VA*	CA*	
FW	1.21 ± 0.03	0.07 ± 0.01	0.13 ± 0.01	0.75 ± 0.01	0.09 ± 0.01	0.07 ± 0.00	1.59 ± 0.03	5.3 ± 0.0
CKM	4.34 ± 0.16	0.84 ± 0.02	0.15 ± 0.01	0.75 ± 0.02	0.23 ± 0.01	0.02 ± 0.01	0	6.2 ± 0.0

*AA: acetic acid, PA: propionic acid, iBA: isobutyric acid, BA: butyric acid, iVA: isovaleric acid, VA: valeric acid, CA: caproic acid.

For the fractionation of each effluent, various two-stage adsorption scenarios were applied, as presented in Figure 1. Each scenario was designed based on the results of screening experiments and the distribution of available VFAs. In each scenario, the objective of the first adsorption stage was to separate the higher MW VFAs from the lower ones, while in the subsequent stage, the focus was to adsorb all remaining VFAs except AA. In each stage, batch adsorption experiments were conducted in a rotary shake incubator (200 rpm and 25 ± 2°C). Fractionation of the FW was conducted using two scenarios. In both scenarios, the first step involved applying GAC-Unwashed at the initial pH of the effluent. For the second step, two different approaches were employed: a) repeating the first step (i.e. using GAC-Unwashed with no adjustment of initial pH) to examine the capacity of the adsorbent to fractionate the remaining VFAs and b) employing the DI-MQ Washed at an adjusted pH of 3.5 to maintain AA in the solution. For the fractionation of CKM, three scenarios were implemented as follows: **1)** Step-1: GAC-Unwashed (at initial effluent pH, i.e. 6.2 ± 0.0), Step-2: GAC-Unwashed (No pH adjustment), **2**) Step-1: GAC-Unwashed (at initial effluent pH), Step-2: DI-MQ washed at an adjusted pH of 3.5, **3**) Step-1: DI-MQ washed at an adjusted pH of 3.5, Step-2: DI-MQ washed (No pH adjustment).

Initial effluents were pretreated before being subjected to the adsorbent in the first stage. Initial CKM effluent was pretreated by centrifugation at 14,800 rpm for 15 min, followed by filtration of the supernatant through 0.2 μm syringe filter to eliminate the remaining microorganisms and suspended solids. However, the initial FW effluent was only pretreated by filtration due to containing fewer suspended particulates. The initial total adsorbate concentrations in the first stage for FW and CKM were 3.91 and 6.33 g L^−1^, respectively. Experiments were conducted in 250 mL Erlenmeyer flask containing 80 mL of VFAs mixtures. The adsorbent dosage in the first stage for FW and CKM were 97.75 and 158.25 g L^−1^, respectively. The adsorbent dosage-to-adsorbate initial concentration ratio was maintained at 25 (g L^−1^ per g L^−1^) in each stage. Each adsorption step lasted 2 h (sufficient contact time for achieving adsorption equilibrium). Liquid-phase samples were collected at intervals of 10, 20, 30, 60, and 120 min and filtered through 0.2 µm syringe filter to monitor their respective pH and VFA concentrations. After each step, the adsorbents were separated from the liquid media by applying filter paper (pore size < 10μm). These sets of experiments were performed in duplicate, and each set included a control flask excluding the presence of the adsorbents.

### 2.3. Analytical methods

The concentration of VFAs was analyzed utilizing the Gas Chromatography (GC) instrument (Clarus 590, PerkinElmer, Shelton, CT, USA) equipped with a flame ionized detector (FID) and a capillary column (Elite-Wax ETR, 30 m × 0.32 mm × 1.00 µm, PerkinElmer, Shelton, CT, USA). Nitrogen gas served as carrier gas at a pressure of 8.27 bar and a flow rate of 12 mL min^−1^. The initial oven temperature was set at 40°C and raised to 200°C at a rate of 10°C min^−1^. The temperature of the injector and detector was fixed at 250°C. Preparation of the samples for GC-FID analysis was conducted as follows: samples were centrifuged (14800 rpm for 5 min), and subsequently, 1 mL of supernatant was mixed with 200 µL acid mix solution (composed of 25% (v/v) ortho-phosphoric acid and 25% (v/v) formic acid at a ratio of 3:1) to protonate the -COOH groups of the VFAs, for increased volatility and to precipitate other available organic compounds. After vortexing, the mixture was centrifuged (14800 rpm for 5 min) and filtered through a 0.2 µm syringe filter to eliminate suspended particles. The final step involved combining 250 µL of supernatant with 250 µL butanol (1 gL^−1^) as an internal standard and 500 µL milli-Q water for GC analysis. In each adsorption experiment, the pH was measured using the Mettler Toledo electrode (LE422, OH, USA), and pH meter (F20 Five Easy, OH, USA).

### 2.4. Data analysis

The adsorption percentage and selectivity were calculated based on Equation 1 and 2 [22]:

(1) $$\mathrm{Adsorption}\%=\frac{C_{e}-C_{0}}{C_{0}}\times 100$$

Where C_0_ and C_e_ are the initial and equilibrium adsorbate concentrations in the liquid phase, respectively.

(2) $$\mathrm{Selectivity}=\frac{y_{i}}{y_{j}}/\frac{x_{i}}{x_{j}}\times 100$$

Where *x* is the mole fraction of the liquid phase in the initial solution, *y* is the mole fraction of the adsorbed phase at equilibrium, and subscripts *i* and *j* denote the respective components.

Statistical analysis of the experimental data was performed using Minitab® 21.1.1 (© 2022 minitab, LLC, USA). Paired t-test analysis was used for the comparison of groups of the data, considering the level of significance as 0.1.

## 3. Results and discussion

In the current study, the suitable adsorbents for VFA fractionation with the optimum performance in terms of adsorption percentage and selectivity were screened through batch adsorption experiments in a synthetic VFA mixture. Screening experiments for two adsorbents, including activated charcoal (powdered and granular) and Diaion HP-20 resin, were conducted by applying different pre-adsorption washing and subjecting them to synthetic VFA mixture with two initial pH levels, a) below the $pK_{a}$ and b) above the $pK_{a}$ of the mixture. Finally, the efficacy of the screened adsorbents was examined for fractionation of two distinct synthetic and acidogenic fermentation effluents using a two-stage adsorption approach.

### 3.1. Adsorbent screening

#### 3.1.1. Effect of pre-adsorption washing of adsorbents

##### 3.1.1.1. Activated charcoal

**Figure 2.**
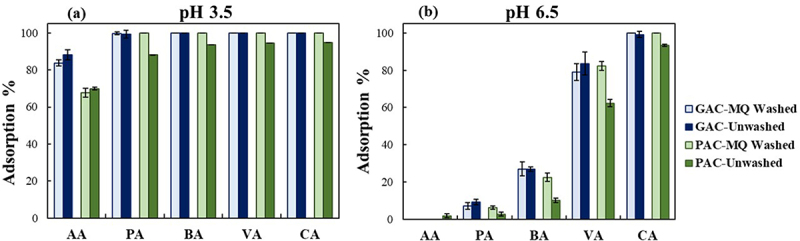
Equilibrium adsorption percentage of MQ washed and unwashed granular and powdered activated charcoal (GAC and PAC) at two initial pH of 3.5 (a) and 6.5 (b).

The effect of washing PAC and GAC with milli-Q water on the adsorption percentage showed a more pronounced impact on PAC compared to GAC, as presented in (Figure 2). Statistical analysis performed on the data of Figure 2, indicated a significant difference between the equilibrium adsorption percentage of washed and unwashed PAC (P-value = 0.011), while the adsorption behavior of washed and unwashed GAC remained identical (P-value = 0.162). The unaffected adsorption performance of the GAC after washing can be attributed to its extra purity. This may also be the cause of the lower adsorption of PAC-Unwashed, with total adsorption percentage of 88.3% and 34.0% at pH 3.5 and 6.5, respectively, compared to the performance of GAC-Unwashed, which exhibited the highest total adsorption percentage of 98.0% and 44.4% at both pH 3.5 and 6.5, respectively. Thereby, the lowest adsorption at both pHs compared to other alternatives belonged to PAC-Unwashed; however, when comparing the selectivity of the studied adsorbents, this particular adsorbent exhibited the highest selectivity (Table 2).

**Table 2.** Selectivity of unwashed powdered and granular activated charcoal (PAC and GAC) versus the milli-Q water (MQ) washed ones at two distinct pH of 3.5 and 6.5.

	Selectivity over CA								
	pH = 3.5					pH = 6.5			
	PAC		GAC			PAC		GAC	
	MQ washed	Unwashed	MQ washed	Unwashed		MQ washed	Unwashed	MQ washed	Unwashed
AA*	0.68	0.74	0.84	0.88		0	0	0	0
PA*	1	0.93	1	0.99		0.06	0.02	0.07	0.09
BA*	1	0.99	1	1		0.23	0.11	0.27	0.27
VA*	1	1	1	1		0.82	0.67	0.79	0.84
CA*	1	1	1	1		1	1	1	1

*AA: acetic acid, PA: propionic acid, BA: butyric acid, VA: valeric acid, CA: caproic acid.

**Figure 3.**
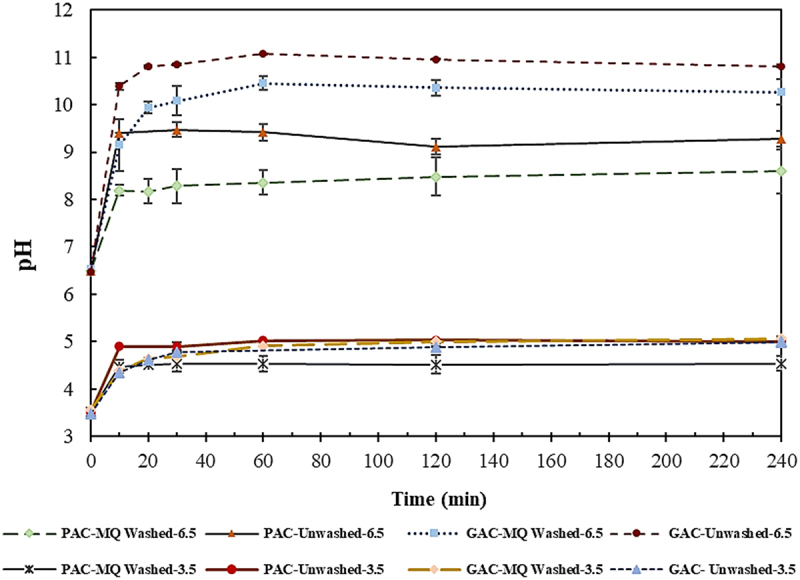
Variation of pH over time for MQ washed and unwashed granular and powdered activated charcoal (GAC and PAC) at two initial pH of 3.5 and 6.5.

Monitoring the pH variations during the adsorption process revealed an immediate rise in pH upon the addition of the adsorbents to the solution indicating the prompt initiation of the adsorption process. As shown in Figure 3, the pH continued to increase until reaching the plateau similar to the trend of adsorption percentage over time (Figures S1 and S2). The magnitude of the changes in pH were more pronounced at 6.5 compared to 3.5. Moreover, a statistical analysis conducted on the pH data presented in Figure 3 showed that washing meaningfully reduced the pH at each contact time for both powdered (P-value = 0.001) and granulated form (P-value = 0.001) at pH 6.5; however, at pH 3.5, the significant reduction of pH only occurred for powdered form (P-value = 0.000) while there was no difference between the pH of washed and unwashed GAC (P-value = 0.706). This is in line with the observed adsorption behavior at both pHs (Figure 2) and supports the hypothesis that more active sites are available by the washing process as a result of the dissolution of the available residual impurities. In addition, according to Figure S1, adsorption equilibrium was achieved for all the VFAs within 10 min when utilizing PAC, while as evident in Figure S2, the process took varying duration for the adsorption of different VFAs to GAC. This is in agreement with the general lower adsorption rate of GAC compared to PAC that has been previously reported [43,44].

##### 3.1.1.2. Diaion HP-20 resin

**Figure 4.**
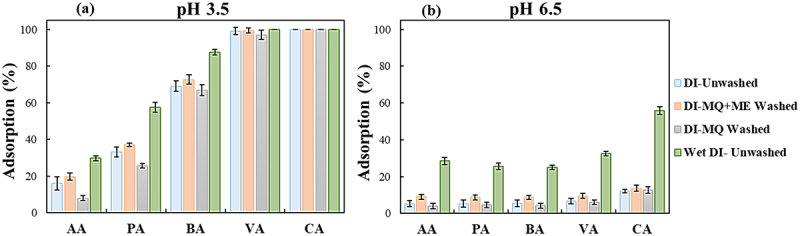
Equilibrium adsorption percentage of washed and unwashed Diaion HP-20 resin (DI) at two initial pH of 3.5 **(a)** and 6.5 **(b)**.

The impacts of different washing methods on DI adsorption performance were investigated and compared with the equilibrium adsorption percentage of unwashed wet and dry DI, as shown in Figure 4. A general look at the results shows the ranking of the studied DI in terms of adsorption percentage as follows: Wet DI-Unwashed>DI-MQ+ME Washed>DI-Unwashed≥DI-MQ Washed. As a result, at pH 3.5, the highest and lowest total adsorption percentages were 75.9% and 66.2%, respectively. Whereas at pH 6.5, the corresponding values were 17.5% and 6.3%. The equilibrium occurred for all the conditions in less than 60 min. The statistical comparison conducted on the data presented in Figure 4 also confirmed the above observation (Table S1). However, when comparing their selectivity, DI-MQ Washed exhibited the highest selectivity with the lowest AA removal (8.0±1.4%) from the VFAs mixture at pH of 3.5 (Table 3). This indicates the potential of DI-MQ Washed to retain the lower MW VFAs in the solution. Eregowda et al. have reported similar results using Diaion HP-20 resin without any pretreatment in a synthetic mixture of VFAs containing 2–5 carbon atoms at the corresponding $pK_{a}$ value of the VFA mixture [20]. However, the adsorption percentage of all studied VFAs was lower than the results obtained in the present study at a pH of 3.5. This disparity can be attributed to the difference in pH of the performed experiments which significantly influence the adsorption percentage that will be discussed further in section 3.1.2.

**Table 3.** Selectivity of washed and unwashed Diaion HP-20 resin (DI) at two initial pHs of 3.5 and 6.5.

	Selectivity over CA								
	DI								
	pH = 3.5					pH = 6.5			
	Unwashed	Wet-Unwashed	MQ** Washed	MQ** +ME*** Washed		Unwashed	Wet-Unwashed	MQ** Washed	MQ**+ME*** Washed
AA*	0.16	0.30	0.08	0.20		0.44	0.51	0.31	0.66
PA*	0.33	0.58	0.26	0.37		0.44	0.46	0.36	0.64
BA*	0.69	0.88	0.67	0.73		0.46	0.45	0.33	0.63
VA*	0.99	1.00	0.97	0.99		0.55	0.58	0.48	0.69
CA*	1.00	1.00	1.00	1.00		1.00	1.00	1.00	1.00

*AA: acetic acid, PA: propionic acid, BA: butyric acid, VA: valeric acid, CA: caproic acid.

**Milli-Q water.

***Methanol.

**Figure 5.**
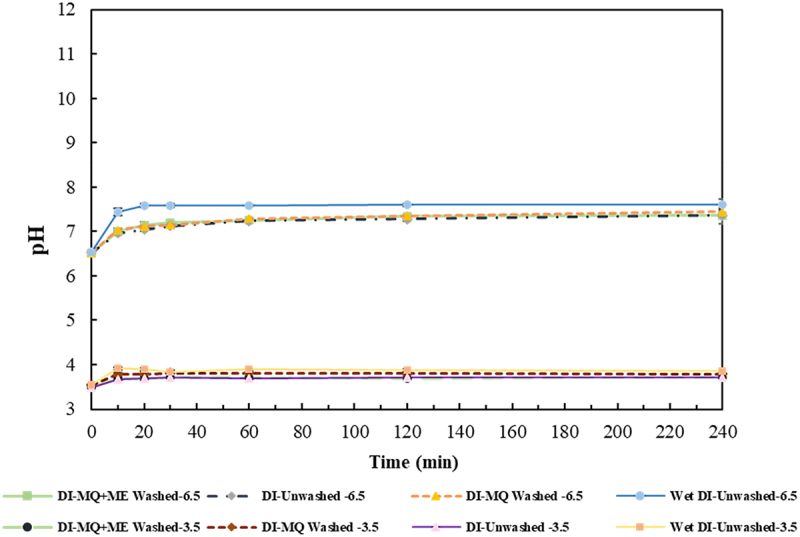
Variation of pH over time for washed and unwashed Diaion HP-20 resin (DI) at two initial pH of 3.5 and 6.5.

The variation in pH over time under different investigated conditions is shown in Figure 5. Meaningfully higher pH at each contact time was observed when employing Wet-DI compared to other studied conditions (P-value≤0.001) which follow a similar pattern to their adsorption behavior (Figure S3). This is also consistent with the findings of activated charcoal as explored in section 3.1.1.1. This implies that the pH variation can be used as an indicator of the adsorption pattern in a VFA mixture. Additionally, the comparatively lower adsorption capacity of DI than activated charcoal can be ascribed to its smaller surface area of ∼500 m^2^ g^−1^, compared to 800–1500 m^2^ g^−1^ associated with activated charcoal.

#### 3.1.2. Effect of initial pH

The experiments carried out at two distinct initial pHs aimed to investigate how the chemical state of VFAs (acid or conjugated base form) influences the adsorption efficiency. The average $pK_{a}$ of a mixture of the five main VFAs was 4.8. At pH of 3.5 ($\mathrm{pH}<pK_{a}$), VFAs remain undissociated, whereas at pH of 6.5, they exist in their dissociated, negatively charged form. Increasing the initial pH from 3.5 to 6.5, shifting from undissociated to dissociated, led to a decrease in the adsorption percentage of all VFAs in the runs using DI (Figure 4). However, the decline in adsorption performance was less pronounced in Wet DI-Unwashed compared to the other studied resins. The smallest performance reduction with increasing pH was observed for AA, while the reduction increased overall with the molecular weight of the acids, resulting in the highest reduction for CA. When applying activated charcoal, a reduction was noticed for all VFAs except CA, which highlights the promising capacity of activated charcoal for targeting and separating the higher MW VFAs (CA and VA) from the lower ones at pH of 6.5 (Figure 2). The average reduction in adsorption percentage for AA, PA, BA, and VA across all the studies with activated charcoal was 76.9%±9.2, 90.4%±3.2, 76.7%±4.2, and 21.8%±6.2, respectively.

The interaction of VFA molecules with Diaion HP-20 resin – nonpolar copolymer styrene-divinylbenzene resin lacking any functional groups – occurs through the hydrophobic interactions between their hydrocarbon chain and the resin surface, as well as hydrogen bond−π interactions between VFA’s carboxyl groups and the aromatic rings of the resin [15,45]. When the pH level rises, the dominance of hydrophobic adsorption prevents the adsorption of VFAs on the resin surface in their dissociated forms [37]. Similarly, the primary adsorption mechanism on activated charcoal surface is driven by hydrophobic interaction [15,23,35,36]. However, the possibility of the existence of inorganic substances and organic functional groups – generated during the production and modification of activated carbon – can result in chemisorption. Therefore, the adsorption of VFAs on activated carbon surface relies either on the porosity of the carbon or on the chemical nature of the surface [46,47]. For both adsorbents, the longer the aliphatic chain of the acids, the higher the hydrophobic interaction and adsorption capacity, which aligns with Traube’s rule [15,23,35,36]. However, the distinct affinity of activated charcoal to CA (100.0%) and VA (≈ 80%) at pH of 6.5, might be due to the stronger negative charge of conjugate base (R-COO^−^) corresponding to higher MW VFAs and their contribution to chemical sorption by binding to acidic groups or cations on the surface of activated charcoal [36], due to the charges in the surface density of as well as the protonation and deprotonation of the functional group of activated carbon in response to pH variation [48].

The high adsorption percentage (Figure 2a) of the activated charcoal at pH of 3.5, in either powdered or granulated form, emphasizes its remarkable capacity for simultaneous and efficient removal of all the VFAs available in the mixture. The lower affinity toward AA is attributed to its hydrophilic nature, as indicated by the negative water-octanol partition coefficients (K_ow_=−0.2) [35]. Earlier research studies have reported the lower adsorption performance of the activated carbon in VFA recovery [35–37]. In one study, however, the majority of AA (88.9%) and BA (98.5%) were adsorbed by activated carbon from fermented leachate in an optimized experiment at a pH value of 3 [24]. The discrepancy in the adsorption behavior of different studies substantially arises from the variation in the origin of the used activated carbons and their production method, which affect the activated carbon surface chemistry, physical properties, pore structure, and impurity level [47].

### 3.2. VFAs fractionation from synthetic and acidogenic fermentation effluents

In the next part of the study, the potential of the screened adsorbents was examined for the VFA fractionation of two acidogenic fermentation VFA effluents and their corresponding synthetic simulated media. The selection of the adsorbents for each stage was based on the pH and the concentration distribution of VFAs in the media.

#### 3.2.1. Food waste-derived VFA effluent

##### 3.2.1.1. Synthetic food waste VFA effluent

Two scenarios were applied to fractionate FW VFA mixture as described in section 2.2.3. Given that the average pH of the effluent was 5.3 which is higher than the average $pK_{a}$ of its VFA constituents; the first step in both scenarios included applying GAC Unwashed with demonstrated capacity to selectively remove higher MW VFAs from the lower ones at pH > $pK_{a}$ (Figure 2b). It is worth noting that GAC was preferred over PAC due to its relatively higher adsorption percentage, cost-effectiveness, absence of dust pollution, and lack of separation issues [43].

**Figure 6.**
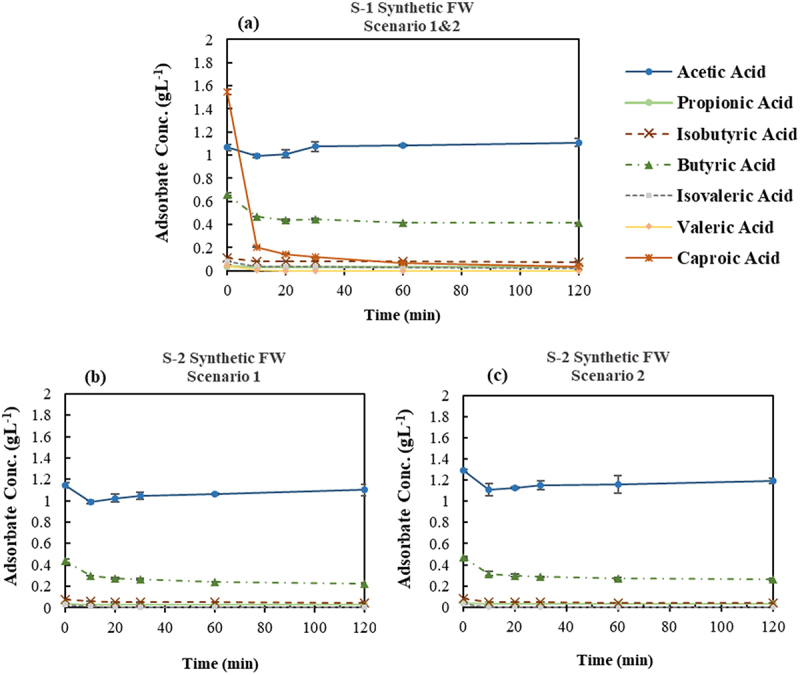
Variation of VFAs concentration over time in synthetic food waste VFA effluent applying two different scenarios, (a) Step-1 of both scenarios using GAC-Unwashed-No pH adjustment, (b) Step-2 of the first scenario using GAC-Unwashed-No pH adjustment, (c) Step-2 of the second scenario using DI-MQ Washed at adjusted pH of 3.5.

The variation in VFAs concentration in the synthetic FW VFA effluent under two scenarios is shown in Figure 6. According to Figure 6a, applying GAC-Unwashed as the first adsorption stage in both scenarios resulted in a significant removal of CA by 97.8%±0.4 while leaving AA unchanged in the solution which is in accordance with the results obtained in the screening experiment. The concentration of the BA decreased from 0.66±0.01 gL^−1^ to the average equilibrium concentration of 0.42±0.01 gL^−1^, representing 37.0%±0.7 of the adsorption percentage. Simultaneously, other VFAs with initially low concentration in the solution reached a final concentration of ≤0.08 gL^−1^. It should also be noted that the pH increased with the same trend as the adsorption percentage and reached the plateau at pH value around 9.6±0.2. The concentration of the VFAs at the end of the initial step represents a solution primarily consisting of AA and BA, pointing to the efficacy of the first step to fractionate the synthetic FW VFA effluent; while the adsorbed VFAs on GAC-Unwashed were mainly comprised of CA and BA with negligible amount of other VFAs.

Relatively similar results were obtained in the second step for both scenarios in synthetic FW VFA (Figures 6b,c). The concentration of AA remained almost constant in the solution while the average removal of the BA by the different adsorbents employed in the second step of the scenarios was 45.4%±2.5. The adsorption percentage of BA can be increased further by repeating the cycle or increasing the adsorbent-to-adsorbate ratio to higher than 25. The pH trends in the second step showed an increase to 10.3±0.0 for GAC-Unwashed, whereas in the case of DI-MQ Washed with the initial pH of 3.6±0.0, there was a slight rise to 3.7±0.0. It is in line with the findings in section 3.1.1. The pronounced pH shift observed with GAC-Unwashed is due to the reduced buffering capacity of the VFA solution at the initial pH of the experiment with GAC-Unwashed (i.e. 8.2±0.1), which was significantly higher than $pK_{a}$ of the solution [49].

##### 3.2.1.2. Food waste acidogenic fermentation VFA bearing effluent

**Figure 7.**
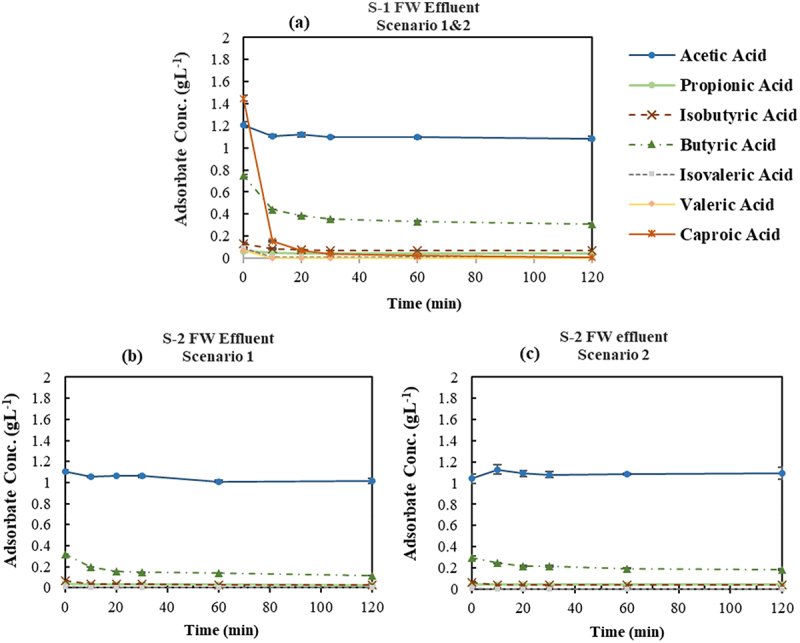
Variation of VFAs concentration over time in acidogenic fermentation VFA effluent derived from food waste applying two different scenarios, (a) Step-1 of both scenarios using GAC-Unwashed -No pH adjustment, (b) Step-2 of the first scenario using GAC-Unwashed -No pH adjustment, (c) Step-2 of the second scenario using DI-MQ Washed at adjusted pH of 3.5.

A similar procedure to section 3.2.1.1 was employed in order to examine the potential of the applied scenarios on the FW effluent (Table 1) which contains impurities and salts in addition to the VFAs as presented in [34]. The results are demonstrated in Figure 7. The adsorption percentage of the CA in the first step (Figures 7a,b) was 99.4%±0.0; however, contrary to the findings of synthetic FW VFA mixture, there was a slight adsorption of AA (9.0%±1.2) and a higher removal percentage for BA (56.5%±2.2); where the concentration of BA and other minor VFAs reduced to 0.33±0.22 gL^−1^ and ≤0.07 gL^−1^, respectively. In addition, a noticeable rise in pH to 8.1±0.0 was observed. The observed results were generally in accordance with the findings of the synthetic effluent.

The adsorption behavior of the utilized adsorbents in step-2 was almost identical in both scenarios, which is in agreement with the findings from synthetic effluent. Specifically, both GAC-Unwashed and DI-MQ Washed at initial pH of 7.5±0.0 and 3.6±0.0, respectively, could decrease the BA concentration to 0.16±0.04 gL^−1^ while keeping the AA concentration practically intact in the solution (Figures 7b,c). Comparing the final concentration of BA in FW effluent with synthetic one at both steps reveals a variance of ≤0.1 gL^−1^, however, it is not significant within the scope. Moreover, the final observed pH for GAC-Unwashed and DI-MQ Washed were 8.6±0.0 and 3.7±0.0, respectively. The lower pH values obtained at each contact time compared to synthetic effluent might be attributed to the higher buffering capacity of the acidogenic fermentation effluent influenced by the presence of other constituents like ammonia and phosphate [42].

Several key findings emerge from the analysis of both synthetic and acidogenic fermentation effluent from FW. First, the adsorption capacity of the utilized adsorbents was comparable in a solution with uneven distribution of VFAs, rich in both lower and higher MW VFAs (i.e. GAC-Unwashed (initial $\mathrm{pH}>pK_{a}$) and DI-MQ Washed (initial $\mathrm{pH}<pK_{a}$)). However, in comparison between GAC-Unwashed and DI-MQ Washed, the former is the superior alternative, requiring no pH adjustment or additional chemical usage with lower purchase cost. The second point is the promising potential of the utilized adsorbents for purifying AA at the end of the second stage. In addition, the adsorbed VFAs in the first stage were primarily composed of caproic acid, which exhibited an impressive adsorption percentage (>97%). This high adsorption of caproic acid was accompanied by adsorption of lower concentrations of lower molecular weight VFAs, suggesting the potential for their selective recovery through a multi-stage desorption method similar to the study of Talebi et al. [24], where their study demonstrated successful recovery of 89.1% of acetic acid using deionized water and 67.8% of butyric acid applying ethanol from activated carbon through a multi-stage desorption process.

#### 3.2.2. Chicken Manure-derived VFA effluent

##### 3.2.2.1. Synthetic chicken manure VFA effluent

**Figure 8.**
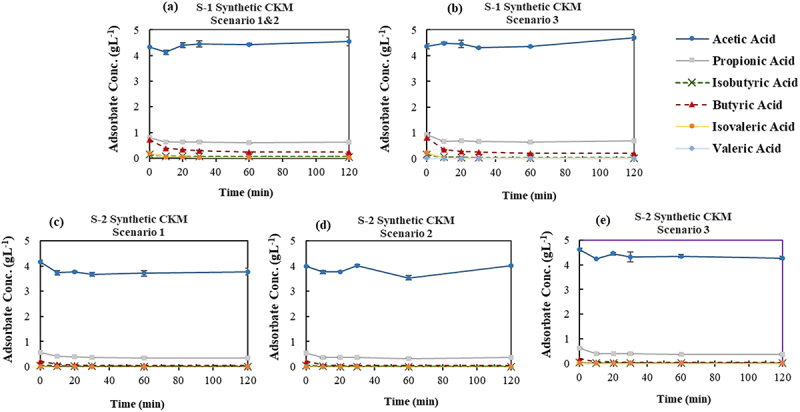
Variation of VFAs concentration over time in the synthetic chicken manure VFA effluent applying three different scenarios. (a) Step-1 of Scenarios 1 and 2: GAC-Unwashed (at initial effluent pH, i.e. 6.2 ± 0.0), (b) Step-1 of Scenario 3: DI-MQ washed at pH 3.5, (c) Step-2 of Scenario 1: GAC-Unwashed (No pH adjustment), (d) Step-2 of Scenario 2: DI-MQ washed at pH 3.5, (e) Step-2 of Scenario 3: DI-MQ washed (No pH adjustment).

Three scenarios were implemented to fractionate the CKM VFA mixture as described in section 2.2.3, based on the results of the screened adsorbent in section 3.1 and the distribution of VFAs in the effluent. Both scenarios 1 and 2 followed a similar approach in their initial step. In each scenario, the primary goal of the initial step was to extract BA and other higher MW VFAs present at lower concentrations, while the subsequent step attempted to mainly remove PA along with other minor VFAs, leaving AA in the solution. The results of multi-stage adsorption of the synthetic CKM VFA effluent are shown in Figure 8. Comparison of the final concentrations of VFAs in the first step of all the scenarios (Figures 8a,b) indicates almost identical behavior of the adsorbents in which nearly all VA, iVA and iBA were removed from the VFA solution, and PA and BA adsorbed with an average removal percentage of 26.1% ± 3, 70.2% ± 5.4, respectively.

In line with the findings from the first step, the diverse conditions employed in the second step of all scenarios led to reasonably analogous results (Figures 8c-e). BA was substantially extracted from the VFAs solution, reaching an average final concentration of ≤0.05 gL^−1^, while PA was partially removed with an average adsorption percentage of 38.2%±2. Moreover, slight adsorption of AA was observed across all scenarios. The least adsorption of AA (≤8%) was observed when employing DI-MQ washed at pH 3.5 (scenarios 2 and 3) which highlights the most effective approach. Consequently, the synthetic solution at the end of the second step in all scenarios was mainly composed of AA (≈4 gL^−1^) with the residual PA (≈0.4 gL^−1^).

##### 3.2.2.2. Chicken manure acidogenic fermentation VFA bearing effluent

**Figure 9.**
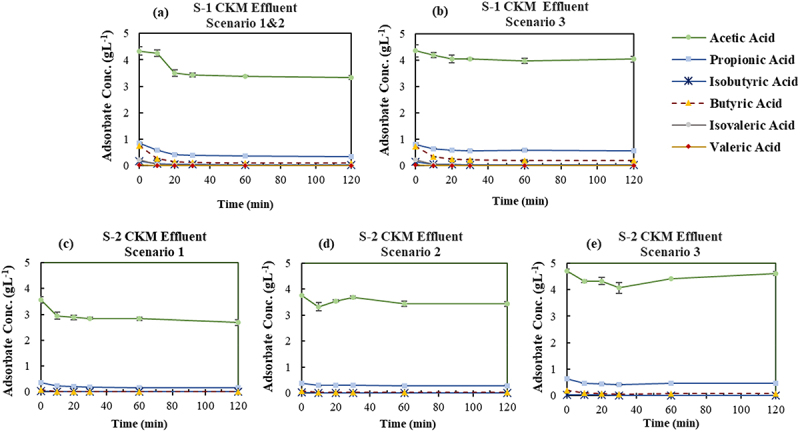
Variation of VFAs concentration over time in the acidogenic fermentation VFA effluent derived from chicken manure applying three different scenarios. (a) Step-1 of Scenario 1&2: GAC-Unwashed (at initial effluent pH, i.e. 6.2 ± 0.0), (b) Step-1 of Scenario 3: DI-MQ washed at adjusted pH of 3.5, (c) Step-2 of Scenario 1: GAC-Unwashed (No pH adjustment), (d) Step-2 of Scenario 2: DI-MQ washed at pH 3.5, (e) Step-2 of Scenario 3: DI-MQ washed (No pH adjustment).

To evaluate the impact of the applied scenarios on the acidogenic fermentation VFA effluent derived from CKM, the same multi-stage adsorption procedure as outlined in section 3.2.2.1 was investigated. As depicted in Figures 9a,b, when the adsorbents were exposed to the CKM acidogenic fermentation VFA effluent in the first step, they exhibited distinct adsorption behaviors from one another in opposition to the observed results with synthetic effluent. In fact, the obtained concentrations at the end of the initial step showed that both adsorbents entirely removed VA, iVA and iBA which is comparable to the findings with synthetic VFA. However, GAC-Unwashed showed higher removal percentages in the initial step compared to the respective experiment with synthetic media as well as the results obtained with DI-MQ washed, with the average adsorption of 82.6% ±3.1, 57.9% ±1.1, and 22.7%±0.9 for BA, PA, and AA, respectively. On the other hand, the adsorption percentages of BA and PA using DI-MQ washed were rather similar to the ones with synthetic media (section 3.2.2.1) without any adsorption of AA. The major drawback of using GAC-Unwashed in the initial step was the elimination of roughly one-fifth of AA.

The results of the various applied scenarios in the second step (Figures 9c-e) also confirmed the different performance of GAC-Unwashed in terms of adsorption against DI-MQ washed. GAC-Unwashed exhibited a partial adsorption pattern for PA and AA, close to the results obtained in the first step, equal to 56.7% ±3.6 and 22.6% ±3.0, respectively. On the other hand, in scenarios 2 and 3, DI-MQ washed extracted PA and AA with lower adsorption percentages of 26.7% ±1.5 and 6.4% ±3.1, respectively. Both adsorbents effectively adsorb the BA by reducing its concentration to ≤0.06 gL^−1^. While VFA adsorption by GAC unwashed was higher in acidogenic fermentation VFA effluent than the observed results in synthetic media, the opposite trend was observed for DI-MQ washed. The fact that GAC unwashed adsorbed AA partially in both steps makes it an unsuitable alternative for VFAs fractionation of the studied acidogenic fermentation VFA effluent from CKM waste. Consequently, among the three scenarios, scenario 3 in which DI-MQ Washed was employed at pH of 3.5 yielded the most favorable outcome. The resulting solution at the end of step-2 consisted of AA and PA with equilibrium concentrations of 4.39 ± 0.22 and 0.45 ± 0.02 gL^−1^, respectively. Further purification of AA from PA can be achieved by repeating the adsorption cycle or utilizing suitable desorption agents during the desorption process. The pH changes in adsorption experiments of both acidogenic fermentation and synthetic CKM VFA effluent with different adsorbents resembled the trends observed with the FW VFA effluent. However, the magnitude of pH variation was smaller because of the stronger buffering capacity of CKM VFA effluent owing to its higher concentration of ammonia and ammonium nitrogen compared to FW VFA effluent [41,42].

The higher adsorption performance of activated charcoal in both acidogenic fermentation effluents than in the corresponding synthetic media can be explained by the presence of ions and molecules in the fermented effluent and their facilitating role in ion bridging [50,51]. Reyhanitash et al. [15] reported mineral acid co-adsorption on functionalized polystyrene-divinylbenzene resins through ion-pair formation with an ion pair of nitrogen. Conversely, no co-adsorption was observed with non-functionalized adsorbent lacking these nitrogen ion pairs, which is similar to the pattern seen with DI in the present study. This signifies the importance of understanding the interactions between adsorbents and the components present in the effluent for further studies.

## 4. Conclusion

This study assessed the potential of Diaion HP-20 resin and activated charcoal in fractionating VFA mixtures from various sources, including synthetic and acidogenic fermentation effluents of chicken manure and food waste. The results indicated that washing only enhanced the adsorption capacity of PAC. pH-dependent adsorption behaviors were observed with unwashed GAC performing best at lower pH ($<pK_{a}$) for total VFA removal and targeting specific acids (VA and CA) at higher pH ($>pK_{a}$), while DI-MQ Washed demonstrated superior selectivity with the lowest AA adsorption at$\mathrm{pH}<pK_{a}$. Two-stage adsorption of acidogenic fermentation effluents revealed that for VFA mixtures rich in both high and low MW VFAs, like food waste, GAC-Unwashed at pH above the $pK_{a}$ fractionated the higher MW VFAs from the lower ones. Conversely, for mixtures abundant in lower MW VFAs, like chicken manure effluent, reducing the pH below the $pK_{a}$ and employing DI MQ-Washed yielded the best result. This study highlights the importance of selecting appropriate adsorbents according to the composition and pH of the VFA mixture to achieve individual acid fractionation. A complete fractionation of adsorbed VFAs can be achieved by integrating each adsorption stage with a selective desorption process.

## Supplementary Material

Supplementary.docx

## Data Availability

The authors confirm that the data supporting the findings of this study are available within the article and its supplementary materials.
