# Supplementary material for: Fractionation of waste-derived volatile fatty acids by multi-stage adsorption using activated charcoal and Diaion HP-20 resin
Source: Bioengineered. 2025 Feb 4;16(1):2458366. doi: 10.1080/21655979.2025.2458366 (PMC11801348; doi:10.1080/21655979.2025.2458366)
Supplement: Supplementary.docx [file KBIE_A_2458366_SM3583.docx]

**Figure S1.** Adsorption percentage variation over time, **(a)** unwashed powdered activated charcoal at pH 3.5, **(b)** MQ washed powdered activated charcoal at pH 3.5, **(c)** unwashed powdered activated charcoal at pH 6.5, **(d)** MQ washed powdered activated charcoal at pH 6.5.

**Figure S2.** Adsorption percentage variation over time, **(a)** unwashed granular activated charcoal at pH 3.5, **(b)** MQ washed granular activated charcoal at pH 3.5, **(c)** unwashed granular activated charcoal at pH 6.5, **(d)** MQ washed granular activated charcoal at pH 6.5.

**Figure S3.** Adsorption percentage variation of Diaion HP-20 (DI) over time, **(a)** MQ washed DI at pH 3.5, **(b)** MQ washed DI at pH 6.5, **(c)** MQ+Methanol washed DI at pH 3.5, **(d)** MQ+Methanol washed DI at pH 6.5., **(e)** Wet DI at pH 3.5, **(f)** Wet DI at pH 6.5, **(g)** Unwashed DI at pH 3.5, **(h)** Unwashed DI at pH 6.5.

**Table S1**. The results of statistical paired t-test analysis of adsorption percentage of different conditionings applied on Diaion HP-20 (DI).

| Compared groups in terms of adsorption% | | P-Value | |
| --- | --- | --- | --- |
|  |  | **pH=3.5** | **pH=6.5** |
| DI-Unwashed | DI-MQ^*^+ME^**^ Washed | 0.059 | 0.001 |
| DI-Unwashed | DI-MQ Washed | 0.069 | 0.119 |
| DI-Unwashed | Wet DI- Unwashed | 0.075 | 0.004 |
| DI-MQ+ME Washed | DI-MQ Washed | 0.057 | 0.007 |
| DI-MQ+ME Washed | Wet DI- Unwashed | 0.082 | 0.008 |
| DI-MQ Washed | Wet DI- Unwashed | 0.063 | 0.003 |

*MQ stands for Milli-Q water.

**ME stands for Methanol.
